# A renewed challenge to electrical bioimpedance: rapid assessment of pathogenic bacteria

**DOI:** 10.2478/joeb-2023-0001

**Published:** 2023-03-02

**Authors:** Eugen Gheorghiu

**Affiliations:** 1. International Centre of Biodynamics, Bucharest, Romania; 2. University of Bucharest, Bucharest, Romania

I was fortunate to meet Professor Herman Paul Schwan a couple of times. Unforgettable scenes took place during the X ICEBI, held in Barcelona back in April 1998. At the end of most presentations, in a gentle and elegant manner, Professor Schwan used to highlight that similar studies (mostly more advanced ones!) were previously performed by himself together with other emblematic figures of the field. Hence gaining new insights into an electrical bioimpedance (EB) topic appears highly challenging if not erroneous.

However, recent technological advancements concerning both instrumentation and data analysis as well as combining EB with other methods led to sensitive quantitative tools to assess bacteria, and the related antimicrobial susceptibility testing (AST), bringing the topic of Rapid Assessment of Pathogenic Bacteria back into a well-deserved spotlight.

The first electrical impedance assay to assess microorganism growth was described at the end of the 19^th^ Century (Stewart, 1899) [1]. The analytical concept relates to an indirect route to appraise the increasing microbial density of a sample by monitoring the electrical conductivity of the growth medium modified by the microbial metabolism.

Since electrical assays lack specificity, EB microbial assessments are generally affected by low throughput, lengthy assays, have to be performed on previously isolated cultures or in conjunction with a method allowing target microbe separation from the sample. High-throughput, automation and specificity are nowadays enabled via immunomagnetic capturing and combination with magnetophoresis and microfluidics.

Microbial identification, and quantitation as well as rapid AST can be achieved based on EB in conjunction with periodic magnetophoresis, as reported by my group [2,3]. Microbial immunomagnetic capture of the target pathogens out of the clinical bio-samples [3] is performed in conjunction with EB measurements to assess the formation of aggregates of magnetically tagged microbes (AMbM) and provide microbial identification, as well.

EB measurements are simultaneously performed at two radiofrequencies chosen to assess the impedance decrement (related to the beta dispersion) of magnetically tagged live microbes to derive the electrical opacity of the specimen as the ratio of impedance amplitude at the higher versus the one at lower frequency (see also [4]).

EB monitoring of the AMbMs periodic magnetophoresis between two electrodes allows quantitative assessment of microbial load (quantitation) since microbial load relates to the weight of the electrode coverage by AMbMs [2].

Bacterial viability is revealed by electrical opacity of the magnetically tagged microbes (membrane integrity is a marker of cell viability). Since susceptibility/resistance to bactericidal antimicrobials directly relates to microbial viability, EB provides an effective tool for cost effective AST assays.

Besides the suitability to multiplexing, allowing AST of various antimicrobials be performed in parallel, such assays allow for rapid measurements since a relevant amount of magnetically trapped microbial cells could be simultaneously analyzed.

Examples based on microfluidic impedance cytometry were recently reported by Hywel’s group [4] in which simultaneous measurements at two radiofrequencies are also deployed to assess the antibiotic induced alteration via cellular 2-D plots showing electrical size vs. electrical opacity.

While the whole (magnetically tagged) bacterial population can be addressed at once, single cell EB analysis cannot be attained due to the limited spatial resolution of the electrical assays, inherently linked to the electrode size.

Thus, another game changer is provided by combining EB and optical methods e.g., by AC electrically modulated light microscopy assays, as the ones highlighted by recent reports [5,6].

These approaches empower EB assays with the spatial resolution needed to allow single cell analysis without the limitations related to electrode size and microelectrode arrays dimensionality. Single-cell resolution is currently limited to analysis of cells adhered to the sensing electrode, yet access to (the dynamics of) of both electrical and the complementary optical parameters of cellular structures is expected to provide new insights into living cell machinery. These advancements are expected to open up renewed challenges to Electrical Bioimpedance supporting countless applications of Rapid Assessment of Pathogenic Bacteria, so exciting to witness and contribute to.
